# Population Variation of the Fall Armyworm, *Spodoptera frugiperda,* in the Western Hemisphere

**DOI:** 10.1673/031.007.0501

**Published:** 2007-01-25

**Authors:** Pete L. Clark, Jaime Molina-Ochoa, Samuel Martinelli, Steven R. Skoda, David J. Isenhour, Donald J. Lee, Jeffrey T. Krumm, John E. Foster

**Affiliations:** ^1^ University of Nebraska Department of Entomology, 312 F Plant Industry Building, Lincoln, NE, 68583 0816, USA; ^2^ Facultad de Ciencas Biológicas y Agropecuarias, Universidad de Colima Apartado postal no. 36, Tecomán, Colima 28100, Mexico; ^3^ Laboratório de Resistência de Artrópodes a Pesticidas (ESALQ/USP), Av. Pádua Dias, 11 Caixa Postal 09, CEP: 13418-900, Piracicaba-SP, Brazil; ^4^ USDA-ARS-SPASRU Screwworm Research Unit, Panama City, Republic of Panamá; ^5^ Monsanto Company 800 North Lindbergh Blvd., St. Louis, MO, 63167, USA; ^6^ University of Nebraska Department of Agronomy, Lincoln, NE 68583 - 0915, USA

**Keywords:** fall armyworm, molecular genetics, AFLP, AMOVA

## Abstract

*Spodoptera frugiperda* (J.E. Smith) (Lepidoptera: Noctuidae), the fall armyworm is the most economically important maize pest in the western hemisphere. This research focused on the genetic variability of the maize host strain because there is a lack of information in this area of *S. frugiperda* research. Amplified fragment length polymorphism (AFLP) was used to assess the genetic variability of *S. frugiperda* over a large geographic area. Twenty populations were collected from the maize, one population was collected from princess tree, one population was collected from lemon tree, and one population was collected from bermudagrass. The 23 populations were from Mexico, the continental United States, Puerto Rico, Brazil, and Argentina. The objective of this research was to evaluate whether the majority of genetic variability was within populations or between populations. The AFLP results showed that the majority of the genetic variability is within populations and not between populations, indicating minor gene flow and suggesting that *S. frugiperda* in the Western Hemisphere are an interbreeding population.

## Introduction

The fall armyworm, *Spodoptera frugiperda (J.E.* Smith) (Lepidoptera: Noctuidae) is a migratory pest endemic to the Western Hemisphere that occurs from Southern Canada to Argentina (Johnson 1987). *S. frugiperda* is phytophagous and causes considerable economic losses in several important crops such as maize, sorghum, rice, cotton, alfalfa, forage grasses, and occasionally other crops in most of the countries of its range (Sparks 1986). *S. frugiperda* was considered a single species but, since about two decades ago, two morphologically indistinguishable host strains have been identified (Pashley 1986); one strain feeds primarily on maize and sorghum (corn strain), and the other strain feeds on rice and bermudagrass (rice strain) (Pashley et al. 1985; Pashley 1986). The fact that no consistent morphological differences have been found between the two strains, suggests that the strains are very closely related and probably of recent origin (Veenstra et al. 1995). Host strains also exhibit differences in development and survivorship when fed on varieties of bermudagrass (Pashley et al. 1987). They also show reproductive incompatibility (Pashley & Martin 1987), reproductive isolating mechanisms (Pashley et al. 1992), behavioral and physiological factors such as food consumption, utilization, and detoxification enzyme activities (Veenstra et al. 1995), and developmental and reproductive traits when fed on different host plants (Pashley et al. 1995), suggesting that the rice strain larvae were more specialized and affected by their host plant than larvae in the corn host strain.

A rapid PCR based method has been described for detecting large tandem repeat clusters of the *S.* *frugiperda* rice strain sequences from individual adult moths (Nagoshi and Meagher 2003), and mitochondrial markers have been described for identifying *S. frugiperda* host strains (Meagher & Gallo-Meagher 2003). Most of these studies have been carried out with *S. frugiperda* populations from the Caribbean basin and the Southeastern United States collected from rice and maize, but there is a lack of knowledge of the genetic variation in large-scale geographic populations of *S. frugiperda* collected from maize. Pashley et al. 1985 used four Mexican geographic populations collected from maize. Three of the four populations were collected in proximity to the Gulf of Mexico: Tampico Tamulipas, Tuxpan Veracruze, and Campeche Campeche; and one population collected at Arriaga Chiapas located at the southeastern coast of the Pacific Ocean. Other *S. frugiperda* populations were from southern Florida, Louisiana, Texas and Puerto Rico. They found that the genetic differences were largely due to a highly divergent Puerto Rican population collected from rice, all other collections were from maize. Additionally, geographic populations of *S. frugiperda* from Mexico collected from maize exhibited biological differences such as developmental rate, reproductive compatibility, and susceptibility to chemical and biological pesticides according to Lopez-Edwards et al. 1999.

AFLP markers were originally developed for plants with small genomes but also became highly effective for plants with larger genomes. AFLP is a tool that generates highly polymorphic molecular markers that aid in building linkage maps and assessing genetic variation. AFLP has been successfully used for the study of insects (Hawthorne 2001; Parsons & Shaw 2002; Reineke et al. 1999; Takami et al. 2004; Tan et al. 2001; Timmermans et al. 2005), including *S. frugiperda* (McMichael & Prowell 1999; Busato *et al.* 2005).

AFLP is based on selective PCR amplification of restriction fragments from a total digest of genomic DNA. The amplification is achieved by using adapted and restriction site sequences as target sites for primer annealing. Selective amplification is achieved by amplifying fragments that have primer extensions that match nucleotides flanking the restriction sites, which allows sets of restriction fragments to be visualized by PCR without prior knowledge of the nucleotide sequence (Vos et al. 1995). A drawback of AFLP is that the procedure may require large amounts of DNA and the process requires multiple operations (Hoy 2003). In the specific case of this research, *S. frugiperda* are relatively large and provided the amounts of DNA needed. AFLP products are sorted according to size using gel electrophoresis. Two types of gels are used: agarose gels may be used to separate fragments from 300 base pairs (bp) to 1 kilobase (kb) pairs in size; and polyacrylamide are the best choice for smaller fragments (10 bp to 20 kb) (Dowling et al. 1996).

The objective of this research was to assess the genetic variability of *S. frugiperda* populations over a large geographic area. AFLP markers were selected to measure genetic variability between 20 *S. frugiperda* populations collected from maize *(Zea mays),* one population collected from lemon tree *(Citrus limon),* one population collected from princess tree *(Paulownia tomentosa),* and one population collected from bermudagrass *(Cynodon dactylon).*

**Figure 1.**
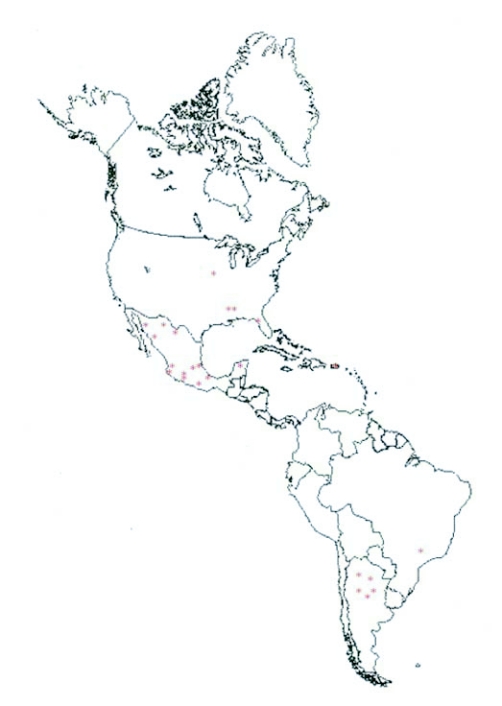
Map of the Western Hemisphere, red asterisks represent the approximate location of a collection.

## Materials and Methods

Samples were collected from each location (Table 1, Figure 1). Samples collected were shipped fresh, in 95% ETOH, or lyophilized. A preliminary study was conducted to evaluate lyophilization as a viable method to preserve *S. frugiperda* DNA for future analysis. The results showed that lyophilized *S. frugiperda* larvae yielded amounts of DNA suitable for molecular analysis similar to fresh *S. frugiperda* larvae. Populations were collected from the Southern United States, Puerto Rico, Mexico, Brazil, and Argentina for this study. Larvae were collected from maize during the vegetative to early reproductive growth stage. Larvae were identified as *S. frugiperda* in the field at the time of collection and again at the Insect Genetics Laboratory at the University of Nebraska using morphological characteristics.

**Table 1.**
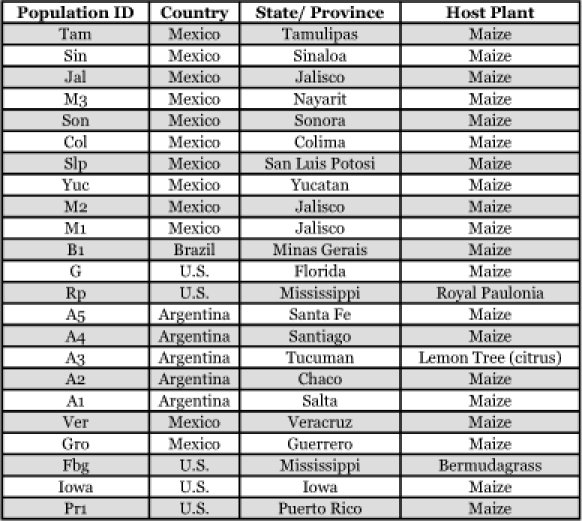
Location and host plant of collected Spodoptera populations, larvae were collected during late vegetative to early reproductive growth stage from maize.

DNA was isolated from seven larvae (7 larvae were used because it was the maximum number of successful extractions for two populations from Argentina, making all populations consistent) per population using a modification of Black & Duteau's (1997) CTAB extraction protocol. Larvae were  collected/preserved using three methods: 1) Fresh and then frozen at -80° C; 2) Larvae collected and placed in vials containing 95% ETOH, then triple rinsed in double distilled H_2_O and stored at -80° C and; 3) Larvae were lyophilized in the country of collection then shipped at ambient temperature and stored dry at -80° C. Larvae were homogenized in 500 µl extraction buffer (100 mM Tris-HCL pH 8.0, 1.4M NaCl, 0.02m EDTA, 2% CTAB, and 0.2% β-mercaptoethanol). Proteinase K (concentration of 200 µg/ml extraction buffer) was added to the homogenate for 2 hrs at 65°C. RNase A (500 µg/ml) was added to the homogenate and held for 3 hrs at 37°C. After RNA and protein were removed from each sample, the homogenate was centrifuged at 13,000 rpm for 5 min at room temperature. The supernatant was extracted with 500 µl of chloroform: isoamyl alcohol (24:1) by centrifugation at 14,000 rpm for 20 min. to separate the phases. The aqueous phase was transferred into an autoclaved 1.5ml micro centrifuge tube and the chloroform: isoamyl step was repeated. DNA was precipitated by adding 400 µl chilled (-20° C) isopropanol to the aqueous phase and incubated at 4°C for 8 h. After incubation the precipitate was centrifuged at 13,000 rpm at 4°C for 30 min. The isopropanol was decanted off; the DNA pellet was rinsed with 500 µl 100% ETOH and centrifuged at 13,000 rpm at 4° C for 5 min and repeated a second time. The ETOH was decanted off and the pellet was air dried at 24°C for 45 min. 50 µl 1X TE buffer (10mM Tris-HCL pH 8.0; 0.1 mM EDTA) was poured onto the DNA pellet and stored at 4°C for 8 hours. After the DNA was re-suspended in 1X TE buffer, each sample was quantified by running a 1% agarose gel with a λDNA marker (22.2 ng/µl). TE buffer (1X) was added to DNA samples until they reached a concentration of 22.2 ng/µl concentration of genomic DNA. The agarose gels were visualized using Genomics Solutions software.

A modified AFLP (Vos et al. 1995) procedure was used as a molecular marker tool to assess the genetic variability of *S. frugiperda* populations. The AFLP procedure was carried out in 3 steps: 1) DNA template preparation; 2) DNA template preamplification and; 3) AFLP Selective amplification

DNA Template Preparation. Restriction digestion was completed using 7 µl of quantified genomic DNA (155.5 ng) that was incubated with EcoRI and *Mse*I restriction endonucleases (New England Biolabs, www.neb.com) for 2.5 hrs at 37°C in solution containing 1.25 µl of 10X One-Phor-All buffer (Amersham Pharmacia biotech, www.apbiotech.com), 0.125 µl of 10 U/µl *Mse*I enzyme (1.25U/reaction), 0.0625 µl of 20 U/µl EcoRI enzyme (1.25 U/reaction), 0.125 µl of 10 ng/ml BSA (bovine serum albumin) (New England Biolabs), and autoclaved nanopure H_2_O. Five µl of ligation mixture containing 0.15 µl of T4 DNA ligase and 0.5 µl of 10X T4 ligase buffer (New England Biolabs), 0.5 µl of 5 pmoles/µl *Eco*RI adapter, 0.5 µl of 5 pmoles/µl MseI adapter (Operon Technologies, www.operon.com), and 3.35 µl autoclaved nanopure water was added to the restriction digestion product and was incubated for 10 hrs at 25°C using an MJ Research (www.operon.com) PTC-200 Peltier Thermo Cycler (used for all PCR reactions); the ligation mixture was diluted by adding 135 µl of 1X TE buffer to each sample.

Preamplification of DNA Template. A master mix of 8 µl pre-amp primer mix II containing two oligonucleotide primers, *Eco*RI adapted ends and *Mse*I ends (Life technologies, www.operon.com), 1.0 µl of 10X PCR buffer containing 15mM MgCl_2_ and 0.20 µl of 5 U/µl Ampli*Taq* DNA polymerase (1.25 U/reaction) (Applied Biosystems, www.appliedbiosystems.com) were added to each reaction tube containing 1 µl of diluted ligation product and amplified using 20 PCR cycles of 30s at 94°C, 1 min at 56°C, and 1 min 72°C. The oligonucleotide primers in the pre-amp primer mix II are complementary to the adapter/restriction site with *Mse*I primer containing one selective nucleotide (M+1 primer) and *Eco*RI primer containing no selective nucleotide (E+o primer). Each preamplification product was diluted with 190 µl of nanopure H_2_O.

AFLP Selective Amplification. Five primers were selected, in four primer pair combinations for selective amplification (Table 2). A selective amplification master mix was made using 2 µl of diluted preamplification product 4.2 µl of autoclaved nanopure water, 1.2 µl of 10X PCR buffer, 0.72 µl 25 mM MgCl_2_, 0.08 µl of 5 U/µl Ampli*Taq* polymerase (Applied Biosystems), 2.0 µl of *Mse*I primer (Invitrogen, www.invitrogen.com) and 0.3 µl of 1.0 pmol/µl IRD-700 labeled *Eco*RI primer (LI-COR, www.licor.com). The “Touchdown” PCR program: 1 cycle of 30s at 94° C, 30s at 65° C, and 1 min at 72° C; 12 cycles of 30s at 94° C, 30s at 65° C (lowering the annealing temperature by 0.7° C) and 1 min at 72°C; 23 cycles of 30s at 94°C, 30s at 56°C, 1 min at 72°C; then hold at 4°C was used for selective amplification. Reactions were stopped prior to loading the selective amplification product on polyacrylamide gels by adding 2.5 µl stop solution (LI-COR) to each reaction tube, then holding at 95°C for 3 min., then cooling to 4° C prior to loading the PAGE gel. 42 samples were run per gel (6 populations of 7 samples each), and IRD-labeled 50-700 bp size marker (LI-COR) in two lanes, 1 µl of marker was loaded into lanes 1 and 44, lanes 2 through 43 contained 1 µl of selective amplification product. Electrophoresis through KB^plus^ 6.5% ready-to-use gel matrix (LI-COR) was used to separate the DNA and the bands were detected by a LI-COR Gene Read IR 4200 sequencer.

**Table 2.**
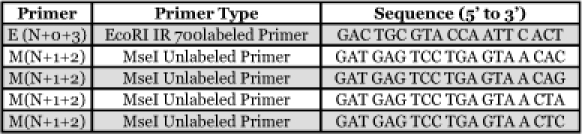
Primers used for selective amplification for AFLP.

AFLP bands were evaluated using an IRD-700 labeled 50-700bp marker as a reference and scored using SAGA Generation 2 Software Version 3.2 (LI-COR, Lincoln, NE). The data set consisted of 1's and 0's (presence or absence respectively) as analyzed from each AFLP gel.

Coefficient of variation is a tool used to assess whether enough data were used (i.e. enough loci, enough primer pair combinations, or both) for proper genetic analysis. DBOOT version 1.1 (Coelho 2001) was used for bootstrap analysis (10,000 iterations) of selected loci, to assess precision for genetic variation and gene flow analysis.

Popgene version 1.32 (Yeh & Boyle 1997) was used to assess the degree of polymorphisms both within and between populations of *S. frugiperda.* Genetic variation (h) between populations was measured using Nei's gene diversity index. Popgene considered a locus polymorphic if the frequency of the most common allele fell below a 0.95 threshold. Genetic variation between populations was assessed using GST. GST is expressed as the heterozygosity (H) of a single population subtracted from the heterozygosity of the total population, divided by the heterozygosity of the total population (G_ST_ = H_Total_ - H_Single_ /H_Total_· Gene flow was estimated from GST, expressed as (Nm) = 0.5 (1- G_ST_) (McDermott and McDonald 1993). Popgene assesses heterozygosity as differences in banding patterns or polymorphisms between individuals or populations.

A binary data matrix was used to estimate genetic similarity using the Jaccard index through the SIMQUAL procedure using NTSYSpc (Rohlf 2000). The similarity coefficient among all populations was estimated using Jaccard:


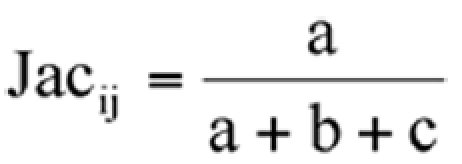


Where:

a = number of cases where a band occurs simultaneously in both individuals

b = number of cases when a band occurs only in the ith individual

c = number of cases where the band occurs on in the jth individual

The Jaccard index was chosen because the coefficient of similarity considers only shared 1s as contributing to the similarity of individuals and disregards shared os. However, there is no general consensus between the various coefficients (Dice, Jaccard, or simple mismatch) for analyzing AFLP fingerprints (Kosman & Leonard 2005).

**Figure 2.**
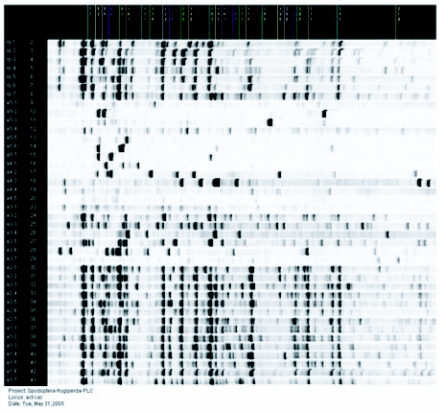
Screen shot of an AFLP gel with markers and lane labels from Saga Generation 2 Software (LI-COR, Lincoln, NE). Note: picture does not represent the length of the full gel, actual gels are 700bp.

Dendrograms were constructed to illustrate genetic similarity, following the methodology described by Sneath & Sokal (1973). Bootstrap analysis was used as a way of testing the reliability of the dataset, by the creation of a pseudoreplicate which re-sampled the data 10,000 times using BOOD-P software ver. 3.1 (Coelho 2001). The goodness of fit of the population dendrogram was measured using COPH and MAXCOMP from NTSYSpc (Rohlf 2000). Analysis of molecular variance (AMOVA) was performed to assess the genetic structure and genetic variability between and among the populations. AMOVA analysis worked by partitioning the total variation between and within populations of *S. frugiperda* and was run using the Arlequin program (Schneider et al. 2000). A two-part AMOVA analysis was performed by testing the genetic structure as a factor of variation among individuals within a given population and between populations. Pairwise comparisons were conducted using the Arlequin program (Schneider et al. 2000) to test genetic divergence (FST). Genetic isolation was tested using the Mantel test with 1000 permutations (Mantel 1967) using NTSYSpc and comparing geographic distance and genetic dissimilarity.

Principle component analysis was run to test genetic isolation using NTSYSpc, and creating an Eigen plot. The principle component analysis is a two-dimensional test that can separate groups into quadrants based on the data. In this analysis, the principal component analysis would give us information regarding genetic isolation among the various populations.

## Results and Discussion

AFLP provided a large number of useable and reproducible markers (Figure 2) to study *S. frugiperda* population genetics. The four primer pair combinations selected to analyze the 23 populations yielded 833 AFLP (56-394 base pairs in size) markers per population (based on population consensus). The primer pair combinations were chosen for their consistency of scorable bands over all populations, polymorphisms, and reproducible banding patterns. Bootstrap analysis of markers indicated that the coefficient of variation of the data (Jaccard index) was 12% explaining 88% of the total variation of the populations, indicating high precision. The coefficient of variation program indicated that the minimum AFLP markers needed per population was 733. Therefore the 833 AFLP markers per population used for the analysis were sufficient for the stability of the UPGMA (unweighted pair group method with arithmetic mean) (Sneath & Snokal 1973) dendrogram. The coefficient of variation test was valuable because the results of the analysis indicated that enough markers were chosen and that primer pair combinations no longer needed to be screened.

The mean genetic diversity (Table 3) within an individual population was at a level that would indicate a lack of homogeneity (HS = 0.2669), which assures more heterozygosity between individuals within a given population resulting in higher diversity needed for physiological and behavioral strength of a population. The total mean genetic diversity over all populations was high (HT = 0.4580) indicating that the entire population has higher genetic diversity than that of individual populations. Both HS and HT values indicate that the level of genetic variability is high between individuals and populations.

**Figure 3.**
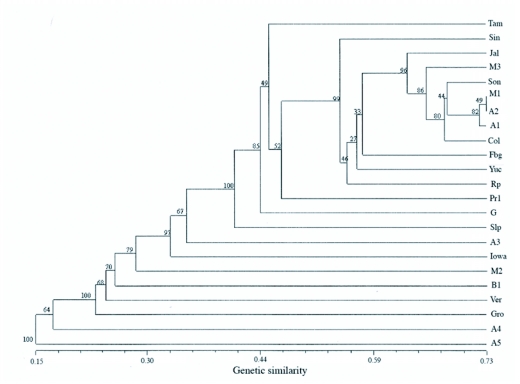
Dendrogram of populations based on similarity coefficient using Jaccard. Bootstrap values are at each node.

**Table 3.**
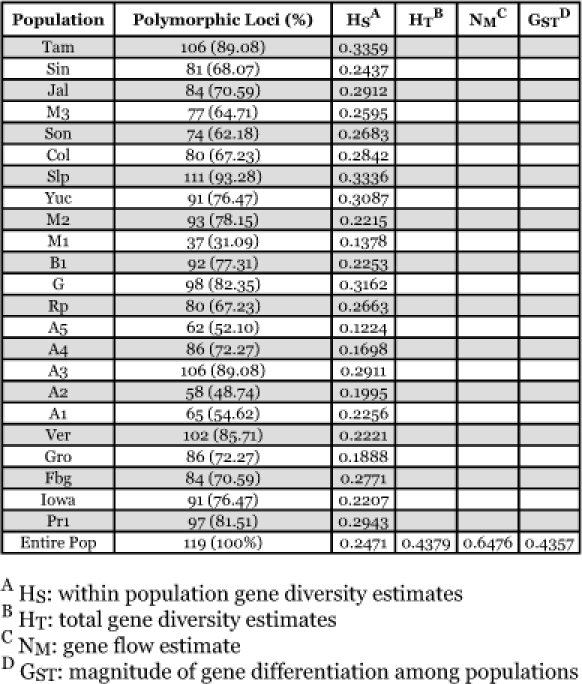
Gene diversity statistics estimated from AFLP data. The individual heterozygosity (Hs) indicates a lack of homogeneity; the total heterozygosity (HT) indicates high genetic diversity of all populations; the gene flow (NM) indicates a low level of gene flow; and the majority of genetic variation is within a given population (GST).

GST measures whether the majority of the genetic variation is within the *S. frugiperda* populations or between the *S. frugiperda* populations. *S. frugiperda* with GST values approaching 1.0 have a majority of genetic variability between populations. Conversely, a lower GST value (< 0.5) infers a majority of the genetic variability is within a given population. The GST value of 0.4172 (Table 3) would indicate that the majority of genetic variability is within given populations *of S. frugiperda* in this study.

Gene flow was measured by using GST values where NM = 0.5(1- GST)/GST (McDermott & McDonald 1993). N is the number of individuals in a population and M is the proportion of individuals that have immigrated into the population. Populations that have an NM value greater than 1, an inference of high gene flow can be assumed. The NM value of 0.6984 (Table 3) shows a low level of migratory gene flow into a given population.

**Figure 4.**
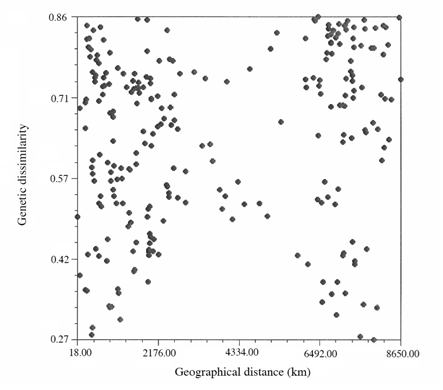
Mantel Test (n = 160, r = 0.1898, p = 0.9455) of genetic dissimilarity (Jaccard) versus geographical distance. The data presented resembles a shotgun blast indicating a lack of correlation between genetic dissimilarity and geographic distance.

The UPGMA consensus tree based on the Jaccard index after bootstrap analysis (Figure 3) shows generally high bootstrap values (BoodP bootstrapping program) at each node of the dendrogram indicating both the reliability of the procedure and the degree of genetic similarity among populations. The UPGMA dendrogram showed that the Argentine population (A5, Santa Fe in maize) was the most dissimilar among populations, while M1 (Jalisco, Mexico in maize) and A2 (Chaco, Argentina in maize) were the most genetically similar (Figure 3). The UPGMA dendrogram did not reveal populations to cluster together solely based on geography (Figure 3). The Jal population clustered with M3, Son, M1, A2, A1, and Col; 98% of the time after 10,000 pseudoreplicates. The overall high bootstrap values indicate confidence in the placement of the populations on the UPGMA dendrogram using the BoodP program.

**Table 4.**
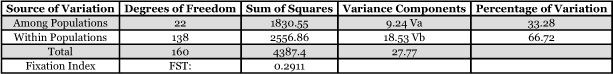
Analysis of Molecular Variance (Amova) Table. The majority of the genetic variation is within a given population, the FST value indicates low gene flow between populations.

The AMOVA results (Table 4) revealed that 33.28% of the genetic variability is between populations. The majority of genetic variability (66.72%) is within given populations. Pairwise comparisons (Arlequin program) of FST = 0.3328 value among all populations confirmed the absence of a geographical associated genetic structure and some genetic isolation.

The genetic isolation by distance was evaluated by a Mantel (1967) test and showed that the matrices representing genetic dissimilarity and geographical distances were not significantly correlated (n = 161, r = 0.1898, p = 0.9455) (Figure 4). These results indicate that there is no correlation between the geographic distance of individuals or populations from each other and the genetic dissimilarity.

**Figure 5.**
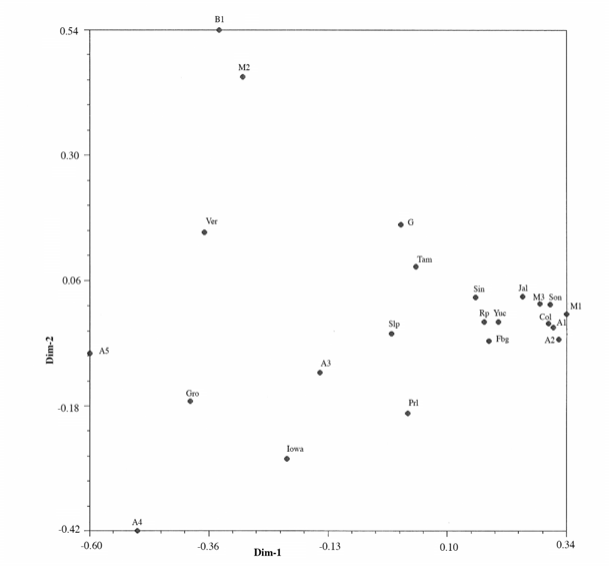
Principle Component Analysis; the clustering was generally non-significant. The data points are random and do not separate into quadrants indicating a lack of genetic isolation. Populations analyzed and presented in the dendrogram and the principle component analysis did not cluster according to the geography or the host plant they were collected from. The lack of population clustering was expected even though it is not consistent with previous research on *S. frugiperda,* due to the high number of populations covering a large geographic area.

Principle component analysis (Figure 5) showed that most populations did not cluster in any consistent location on the graph. However, 11 populations clustered closely but no inferences can be made. Seven of the populations were from Mexico (Sin, Jal, Col, M1, M3, Yuc, Son, collected from maize); 2 populations were from Argentina (A1, A2, collected from maize); 2 populations were from Mississippi (Rp, collected from princess tree, and Fbg, collected from bermudagrass). The majority of the populations were randomly spread out among the graph. The inclusion of very geographically distant populations and lack of separation into quadrants by geographic regions indicates a lack of genetic isolation between the 23 populations.

This work proves that *S. frugiperda* is highly genetically variable and that future studies should incorporate more populations from a large geographic area to obtain more genetic information on host strain, geographic isolation, and reproductive incompatibility. All populations in this study were distinguishable by the selected AFLP markers by calculating a similarity matrix using the Jaccard similarity coefficient. Furthermore, the majority of the genetic variability is within individual populations and not between populations, indicating minor gene flow. This type of molecular research has practical applications for pest control in agriculture. The better we understand the population genetics of this pest, the better we can answer questions about whether or not a control method will work on the entire population or just a segment. Future research of *S. frugiperda* using AFLP and the markers selected from this study could become useful in tracking populations over time.
